# Blood flow disturbances in the central retinal artery in patients with bilateral optic disc drusen

**DOI:** 10.1038/s41598-020-68090-3

**Published:** 2020-07-06

**Authors:** Iwona Obuchowska, Andrzej Ustymowicz

**Affiliations:** 1 Department of Ophthalmology, Medical University of Białystok, Białystok, Poland; 2 Department of Radiology, Medical University of Białystok, Białystok, Poland

**Keywords:** Neuroscience, Diseases

## Abstract

To evaluate retrobulbar hemodynamics in patients with optic disc drusen (ODD) by color Doppler imaging. A prospective study included 52 patients with bilateral ODD and 50 age-matched controls. Blood flow in the central retinal artery (CRA) in both eyes was evaluated. Peak-systolic velocity (PSV), end-diastolic velocity (EDV), and the resistivity index (RI) were measured. Mean values of the blood flow Doppler parameters were compared between the patients with ODD and the controls. Both PSV and EDV in the CRA were significantly lower in patients with ODD than in the controls (*p* < 0.001). The RI in the CRA of ODD patients was significantly higher than in the controls (*p* < 0.001). Women with ODD presented with significantly lower values of PSV and EDV than men (*p* = 0.05 and *p* = 0.03, respectively). Arterial hypertension, cigarette smoking and location of ODD in the left or right eye did not exert a significant effect on the blood flow in the CRA. Blood flow in the CRA is considerably disturbed in patients with ODD. This disturbance might result from mechanical stenosis of the vascular wall caused by the drusen. Color Doppler ultrasound is a useful method in the assessment of retrobulbar hemodynamics in ODD.

## Introduction

Optic disc drusen (ODD) are hyaline-like deposits localized in the prelaminar portion of the optic nerve head. The prevalence of the ODD is estimated at 0.3–2.0%; in 66–92% of the cases, the drusen are found bilaterally^1,2^. Although the pathogenesis of ODD is yet to be understood, according to the most accepted theory, they form due to disturbance in the normal axonal metabolism and a resultant gradual calcification of mitochondria. This results in a rupture of axonal membranes and extrusion of mitochondria into the extracellular space whereby they undergo further calcification and fuse into larger complexes, forming the drusen^3–5^.

A loss of vision occurs very rarely in patients with ODD and are usually associated with the development of vascular complications^6–10^. The most common clinical manifestations of ODD are visual field defects, according to various reports, detected in 24–88% of eyes with the drusen^11–14^. Studies examining the thickness of the retinal nerve fiber layer (RNFL) demonstrated that the changes in the visual field are associated with ganglion cell axon damage. Furthermore, patients with ODD were shown to present with thinning of the peripapillary RNFL, especially in the superior and inferior quadrant, similar to that observed in glaucomatous eyes^14–17^.

Current evidence suggests that optic nerve damage in patients with ODD may result from disturbances in the axonal flow. These disturbances may be a consequence of (1) improper axonal metabolism in the ganglion cells^3,4^; (2) direct compression of the drusen on the optic nerve fibers in the prelaminar part of the scleral canal^1–3,18^; and (3) ischemia of the optic nerve head caused by vascular abnormalities^1,2,6,19^. It is still unclear whether the presence of calcified drusen in the proximity of normal ocular vessels may directly affect retrobulbar hemodynamics. Similarly, little is known about the consequences of the retrobulbar hemodynamics impairment to the optic nerve functioning. The review of literature showed that only one previously published study used Doppler imaging to determine ocular blood flow parameters in patients with ODD^20^. However, retrospective character of this study and small sample size justify further research in this matter.

In this study, we used color Doppler ultrasonography to verify whether the presence of ODD was associated with blood flow disturbances in the central retinal artery (CRA) and to determine a character of the retrobulbar hemodynamic disorders. To the best of our knowledge, this was the first a large prospective study which compare the blood flow parameters in the ocular vessels of patients with ODD and age-matched controls.

## Materials and methods

### Patients

We examined prospectively all adult patients with anomalous optic disc appearance, admitted to the Department of Ophthalmology, Medical University of Bialystok, between May 2010 and January 2018. All study patients underwent basic ophthalmic examination and visual field testing with Medmont Automated Perimeter (Model M700, Medmont International Pty Ltd, Australia). ODD was diagnosed based on the appearance of the optic disc (lumpy border with bright irregular deposits, yellowish-white elevation, full disc without cupping) during indirect slit-lamp biomicroscopy with Volk lens. If the appearance of the optic disc was atypical, the ultimate diagnosis was confirmed with B-scan ultrasonography (EZ 5500A/B, Sonomed, USA), optical coherence tomography (3DOCT-1000, Topcon, Japan), and fluorescein angiography with evaluation of autofluorescence (Kowa VX-10 Fundus Camera, Kowa, Japan). Moreover, basic laboratory parameters, such as complete blood count, coagulation parameters and blood glucose levels, were determined in all the study patients.

The patients were excluded from the study if they had (1) unilateral drusen, (2) intraocular pressure (IOP) greater than 21 mmHg, (3) evidence of other ocular diseases, not associated with ODD, on ophthalmic examination, (4) refractive error >  ± 3.0 diopters, (5) history of intraocular surgeries or laser treatment, (6) systemic conditions, such as diabetes mellitus, cardiovascular diseases and respiratory disorders, and/or (7) blood clotting disturbances. Arterial hypertension was not an exclusion criterion from the study.

The control group was comprised of 50 age- and sex-matched persons, 20 men and 30 women with a mean age of 43.2 years. To be eligible for the study, the controls need to present with good general health; arterial hypertension was the only concomitant disease which did not constitute an exclusion criterion from the control group. All controls had normal visual acuity and did not show any abnormalities on basic ophthalmic examination and perimetry.

## Methods

Color Doppler imaging (CDI) was preceded by the measurement of IOP and blood pressure (BP). All tests were carried out at the same time of the day, between 9:00 AM and 11:00 AM. IOP was determined by means of Goldmann applanation tonometry and corrected for pachymetry value determined using specular microscope, and BP was measured by sphygmomanometry in a sitting position. The list of analyzed parameters included systolic blood pressure (SBP, mmHg), diastolic blood pressure (DBP, mmHg), pulse pressure index (PPI = [SBP-DBP]/SBP) and ocular perfusion pressure (OPP, mmHg). OPP was calculated from the following formula: 2/3 [DBP + 1/3(SBP-DBP)] – IOP.

Blood flow was examined by an experienced investigator (AU) using an ultrasound unit with a 7.5-MHz linear probe (Siemens Elegra, Germany). Blood flow in the CRA was analyzed in both eyes of each subject. The assessment of the eye’s vascular bed was preceded by the examination of the carotid artery (CA) and ophthalmic artery (OA), to exclude blood flow disturbances in these vessels. The examination was carried out with the patient in a recumbent position, after a small amount of ultrasound gel was applied onto the closed eyelids, with the probe positioned gently not to compress the eyeball. The examined vessel was identified based on its position, the direction of blood flow (arterial flow to the probe highlighted with red and venous flow from the probe highlighted with blue) and relation to the eyeball and optic nerve. The CRA was visualized as a fine artery situated in the central part of the optic nerve. Its spectrum was presented together with the spectrum of blood flow in the central retinal vein, which runs within 10–15 mm of the CRA. The blood flow parameters in the CRA were examined at the standard position, approximately 3–6 mm behind the posterior wall of the eyeball at the Doppler angle’s range of 0–20º. This enabled us to attain the maximum Doppler shift values.

The list of analyzed flow parameters included peak-systolic velocity (PSV, cm/s), end-diastolic velocity (EDV, cm/s), and the resistivity index (RI). PSV was defined as the highest value of blood flow during cardiac systole, EDV as the lowest value of blood flow at the end of cardiac diastole, and RI as the quotient of the difference between peak systolic and peak diastolic velocity over peak systolic velocity [RI = (PSV-EDV)/PSV].

### Data analysis

Normal distribution of continuous variables was verified with the Kolmogorov–Smirnov test. Statistical characteristics of continuous variables were presented as arithmetic means, standard deviations (SD), medians and interquartile ranges (IQR). Statistical characteristics of qualitative and discrete variables were presented as numbers and percentages. Student t-test was used for between-group comparisons of normally distributed variables, whereas the variables with distributions other than normal were compared with the Mann–Whitney U-test. The distributions of qualitative and discrete variables were compared with the chi-squared test or the Fisher exact test. The power of the variables the values of which differed significantly or at a threshold of statistical significance (*p* < 0.1) between the patients with ODD and the controls was estimated on a univariate logistic regression analysis. Odds ratios (ORs) for the occurrence of ODD were calculated during the analysis, along with their 95% confidence intervals (CIs). All variables which turned out to be significant or nearly significant (*p* < 0.1) determinants of ODD on the univariate analysis, were included in a single multivariate logistic regression model. Moreover, a receiver operating characteristic (ROC) analysis was carried out to determine the cut-off values of the hemodynamic variables that distinguished optimally between the patients with ODD and the controls. The following characteristics were calculated for each hemodynamic parameter: (1) sensitivity, i.e. the proportion of the patients with ODD, in whom the values of the analyzed variable were above the proposed cut-off value, and (2) specificity, i.e. the proportion of the controls in whom the values of the analyzed variable were below the proposed cut-off value. The accuracy of the analyzed variables was confirmed based on the values of Z-statistic. Moreover, the areas under the ROC curves (AUCs) were calculated, along with their 95% CIs. All calculations were carried out with Statistica 10 package (StatSoft, Tulsa, OK, USA), with the threshold of statistical significance set at *p* ≤ 0.05.

### Ethics approval

This study was performed under approval from the Bioethics Committee of the Medical University of Białystok in accordance with the ethical standards as laid down in the 1964 Declaration of Helsinki and its later amendments or comparable ethical standards. All the subjects gave a written, fully informed consent for the examination and the use of their clinical data for publication.

## Results

A total of 52 patients with bilateral optic disc drusen, among them 22 men and 30 women aged between 29 and 64 years (mean 42.5 years) were enrolled in the study.

Visual acuity impairment (< 6/6 on the Snellen chart) was observed in 7 (13.5%) patients with ODD. The impairment was bilateral in two patients and unilateral in the remaining five. The presence of peripapillary subretinal neovascular membrane was observed in two of these subjects. Thirty-seven (71%) patients had visual field defects in one (n = 21) or both eyes (n = 16).

**Figure 1:**
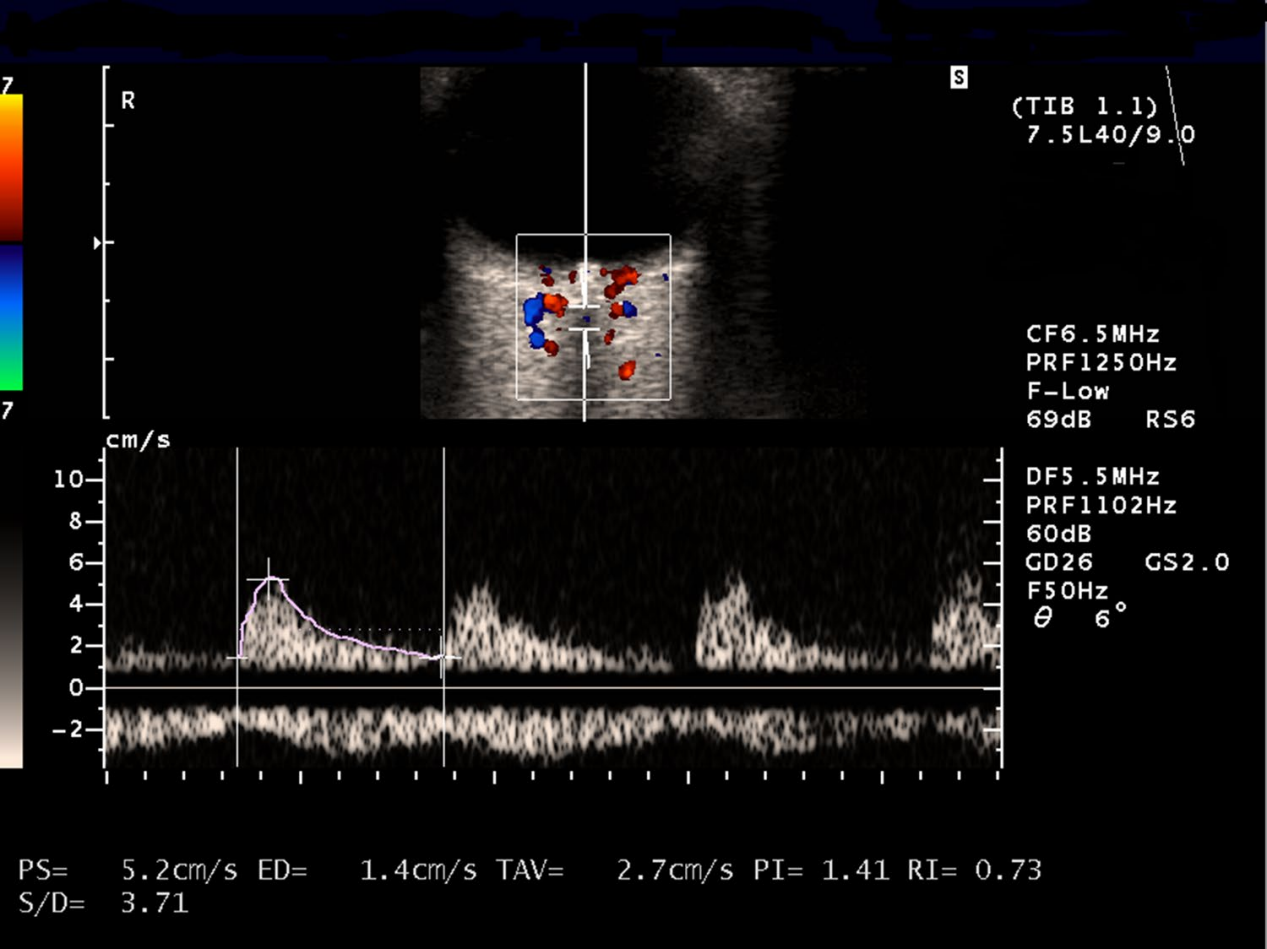
Color Doppler imaging: blood flow spectrum in the central retinal artery in patient with the optic disc drusen—both peak-systolic velocity and end-diastolic velocity are decreased and resistivity index is increased.

The patients with ODD presented with significantly lower values of PSV and EDV in the CRA and significantly higher values of RI than the controls (Fig. 1). No statistically significant between-group differences were observed in terms of age, IOP, SBP and DBP determined at the time of the Doppler imaging, PPI and OPP (Table 1). Moreover, the patients with ODD did not differ significantly from the controls in terms of the distribution of sex, cigarette smoking and arterial hypertension. The ODD group included six current cigarette smokers and seven patients with arterial hypertension. Eight persons from the control group were current cigarette smokers and six suffered from arterial hypertension.

**Table 1:** Statistical characteristics of continuous variables in patients with optic disc drusen and healthy controls.

Variables	ODD (n = 52/104)				Control group (n = 50/100)				*p* Value
	Mean	SD	Median	IQR	Mean	SD	Median	IQR	
Age (years)	42.48	9.08	42	34–50	43.22	8.12	42	38–51	0.541
Peak-systolic velocity (cm/s)	8.94	1.31	9	8.2–9.8	9.51	0.82	9.5	8.9–10	0.000
End-diastolic velocity (cm/s)	2.98	0.66	3	2.65–3.35	3.34	0.48	3.2	3–3.65	0.000
Resistivity index	0.67	0.05	0.66	0.64–0.68	0.64	0.03	0.65	0.63–0.66	0.000
Intraocular pressure (mmHg)	15.81	2.59	15.9	14–17.65	15.81	2.29	15.9	14–17.3	0.992
Systolic blood pressure (mmHg)	131.12	12.22	130	122–137	130.66	12.65	130	122–139	0.794
Diastolic blood pressure (mmHg)	75.85	8.02	75	70–80	76.36	9.32	75.5	70–82	0.673
Pulse pressure index	0.42	0.03	0.415	0.4–0.44	0.41	0.03	0.41	0.39–0.43	0.119
Ocular perfusion pressure (mmHg)	50.8	4.11	50.4	47–54	49.9	3.8	50.1	46–55	0.325

ODD—optic disc drusen, SD—standard deviation, IQR—interquartile ranges.

Univariate logistic regression analysis confirmed that lower values of PSV and EDV in the CRA and higher values of the RI were significant determinants of ODD. However, none of these parameters was identified as an independent determinant of ODD on multivariate logistic regression analysis.

ROC analysis identified the cut-off values for PSV and EDV in the CRA and RI that most accurately distinguished between the patients with ODD and the controls (Table 2). The most sensitive predictor of ODD was PSV below 8.6 cm/s, whereas RI above 0.67 had the highest specificity of all analyzed hemodynamic parameters. However, none of these variables had significantly higher diagnostic accuracy than the others.

**Table 2:** Determinants of optic disc drusen: the results of ROC analysis.

Variable	Value	Sensitivity	Specificity	AUC	95% CI
Peak-systolic velocity (cm/s)	8.6	0.317	0.900	0.632	0.556–0.708
End-diastolic velocity (cm/s)	2.7	0.279	0.950	0.656	0.582–0.730
Resistivity index	0.67	0.087	0.980	0.657	0.582–0.731

ROC—receiver operating characteristic, AUC—area under curve, 95% CI—95% confidence interval.

Compared with male patients, women with ODD presented with significantly lower values of PSV and EDV in the CRA. Patient sex did not exert a significant effect on the values of RI (Table 3). The parameters of blood flow in the CRA were also not significantly modulated by the location of ODD in the right or left eye, smoking and arterial hypertension (Tables 4). No statistically significant correlations were found between the blood flow parameters in the CRA, patient age, IOP, SBP, DBP and PPI (Table 5), and none of the analyzed variables turned out to be a significant prognostic factor of ODD.

**Table 3:** Statistical characteristics of blood flow in the central retinal artery in patients with optic disc drusen, stratified according to sex.

Variable	Woman (n = 30/60)				Man (n = 22/44)				*p* Value
	Mean	SD	Median	IQR	Mean	SD	Median	IQR	
Peak-systolic velocity (cm/s)	8.72	1.51	8.8	7.95–9.8	9.23	0.91	9.4	8.8–9.8	0.050
End-diastolic velocity (cm/s)	2.86	0.74	3	2.5–3.3	3.14	0.51	3.2	2.9–3.4	0.030
Resistivity index	0.67	0.06	0.66	0.645–0.68	0.66	0.03	0.655	0.64–0.68	0.097

SD – standard deviation, IQR – interquartile ranges.

**Table 4:** Statistical characteristics of blood flow in the central retinal artery in patients with optic disc drusen in relation to side of eye, smoking and arterial hypertension.

Variable	Mean	SD	Median	IQR	Mean	SD	Median	IQR	*P* value
	Right eye (n = 52)				Left eye (n = 52)				
Peak-systolic velocity (cm/s)	8.85	1.43	8.9	8.15–9.75	9.02	1.18	9.15	8.4–9.8	0.511
End-diastolic velocity (cm/s)	2.94	0.75	3	2.6–3.3	3.01	0.56	3.1	2.65–3.4	0.556
Resistivity index	0.67	0.06	0.665	0.64–0.68	0.66	0.03	0.655	0.64–0.68	0.493
	Smoker (n = 7/14)				Nonsmoker (n = 45/90)				
Peak-systolic velocity (cm/s)	9.36	1.39	9.05	8.8–9.9	8.87	1.29	9	8.2–9.8	0.197
End-diastolic velocity (cm/s)	3.06	0.54	2.95	2.8–3.2	2.96	0.68	3	2.6–3.4	0.620
Resistivity index	0.67	0.02	0.67	0.66–0.68	0.67	0.05	0.65	0.64–0.68	0.709
	Arterial hypertension (n = 7/14)				No arterial hypertension (n = 45/90)				
Peak-systolic velocity (cm/s)	9.04	1.56	8.85	8.1–9.3	8.92	1.27	9.1	8.2–9.8	0.762
End-diastolic velocity (cm/s)	2.93	0.79	2.95	2.6–3.2	2.98	0.64	3	2.7–3.4	0.779
Resistivity index	0.68	0.05	0.665	0.65–0.68	0.67	0.05	0.655	0.64–0.68	0.431

SD—standard deviation, IQR—interquartile ranges.

**Table 5:** Pearson’s coefficients of linear correlation (r) between the parameters of blood flow in the central retinal artery and other characteristics of patients with optic disc drusen.

Variable	Peak-systolic velocity		End-diastolic velocity		Resistivity index	
	r	*p*	r	*p*	r	*p*
Age	− 0.1464	0.138	− 0.1684	0.088	0.1852	0.060
Intraocular pressure	0.1397	0.157	0.0759	0.444	0.0116	0.907
Systolic blood pressure	0.1227	0.215	0.1034	0.296	− 0.0073	0.941
Diastolic blood pressure	0.0866	0.382	0.0536	0.589	− 0.0093	0.926
Pilse pressure index	0.0421	0.671	0.0783	0.429	− 0.0029	0.977

## Discussion

The aim of the present study was to compare the retrobulbar hemodynamics in patients with ODD and healthy controls. The study demonstrated that the presence of ODD was associated with substantial disturbances of blood flow in the CRA. To the best of our knowledge, only one previous study analyzed the blood flow in the ocular vessels of patients with ODD. Based on the analysis of a small cohort, Pinto et al.^20^ concluded that both patients with ODD (16 eyes) and glaucomatous patients (20 eyes) had lower systolic and diastolic flow velocities in the CRA. The authors did not find statistically significant between-group differences in RI values.

Our study demonstrated that the blood flow in the CRA of patients with ODD had typical features of a resistance flow with disturbed outflow, i.e. (1) a decrease in PSV along with spectrum flattening (changed proportion of systolic wave height to the time of its duration), and (2) marked decrease in EDV and increase in RI. These findings, especially the significantly higher values of RI in ODD patients than in the controls, are indirect indicators of vascular stenosis located peripherally from the examined site^21^. Since the flow measurements in the CRA were taken routinely 3–6 mm behind the posterior ocular wall, the stenosis might have occurred at the level of the prelaminar part of the optic nerve and might result from mechanical compression of the CRA by surrounding drusen. Vascular stenosis usually results in worse perfusion, which in turn leads to ischemia in the area supplied by the affected vessel. As mentioned above, Pinto et al.^20^ did not find significant differences in RI values, which seems surprising given the hereby discussed plausible mechanism of alterations in the CRA flows. This fact was also highlighted by Pinto et al. who mentioned that “the reasons for such results are unclear”^20^.

In our present study, some blood flow parameters (PSV and EDV) of ODD patients were significantly lower in women than in men. However, patient age, location of ODD in the right or left eye, IOP, smoking, SBP, DBP and presence of concomitant arterial hypertension did not exert a significant effect on the blood flow parameters in the CRA of ODD patients. To the best of our knowledge, this study was the first to analyze the effect of clinicodemographic characteristics on the retrobulbar flow, as this issue has not been addressed by Pinto et al.^20^.

Stenosis of small retinal vessels in patients with ODD was also documented in previous studies that used optical coherence tomography angiography (OCTA) to assess the retinal microcirculation. OCTA is a technique to visualize microvessels and to measure their width and density based on the erythrocyte flow. However, OCTA cannot be considered an alternative to the evaluation of hemodynamics by Doppler imaging. The results of OCTA-based studies suggest that the presence of ODD is associated with capillary narrowing in the superficial capillary plexus layer, areas of capillary dropout and decreased vascular density on the optic nerve head^22–25^. These OCTA findings were shown to correlate positively with the RNFL thickness^26^. Based on those findings, it has been concluded that enlarged ODD may cause acute or chronic ischemia by compressing optic nerve fibers and surrounding vessels. Hence, identification of disorders in retinal blood flow, whether on Doppler imaging or OCTA, might be an early predictor of future axonal damage in patients with ODD.

It is still unclear whether the disturbances in the CRA flow may occur secondarily to the optic nerve atrophy and result from reduced metabolic demand in atrophic tissues. Patients with ODD without a doubt show atrophy of the retinal ganglion cell axons^3,4^; however, we still lack objective data regarding the influence of this atrophy on the ocular blood flow. A few previous studies documented vascular changes in the optic nerve atrophy. Some authors found the evidence for the narrowing of total arteriolar and capillary diameter in patients with the optic nerve atrophy^27–29^. Sebag et al.^30^ analyzed blood flow in peripheral branches of the CRA in cats with experimental optic nerve atrophy. Using laser Doppler methods, those authors demonstrated a 53% decrease in erythrocyte speed in the capillaries of the atrophic optic nerve head. Hence, we cannot exclude that the worsening of hemodynamic conditions in the CRA vascular bed might occur secondarily to the atrophy of the optic nerve fibers.

The data gathered during several years of follow-up of patients with ODD suggest that both visual field loss and decrease in RNFL thickness have a progressive character and sometimes may resemble changes associated with glaucoma^2,14,31^. Also, according to Pino et al.^20^, the degree of retrobulbar flow impairment in patients with ODD and glaucomatous patients was similar. No effective treatment to prevent progressive injuries of the optic nerve fibers in patients with ODD has been developed thus far. In a pilot study conducted by Schargus and Gramer^2^, IOP-lowering therapy substantially slowed down the progression of visual field loss in patients with ODD, although the latter have never presented with elevated IOP values. The authors of that study did not fully explain the reasons behind this phenomenon. However, it can be assumed that a decrease in IOP might be associated with an increase in perfusion pressure and resultant enhancement of blood flow through the ocular vessels. Hence, improved retrobulbar hemodynamics associated with a decrease in IOP might exert a positive effect, slowing down the progression of RFNL injuries in patients with ODD, similar as in glaucomatous neuropathy. A better insight into this mechanism could be obtained if the hemodynamic parameters for the vessels supplying the optic nerve were compared between two groups of patients with ODD: those who received an IOP-lowering therapy and those who did not. The results of our present study, as well as the findings reported by Pinto et al.^20^, imply that color Doppler ultrasonography might be a useful diagnostic instrument in this context.

We are well aware of the potential limitations of this study. The most significant constraint pertains to the research methodology. Doppler ultrasonography visualizes solely hemodynamic parameters of flow in a vessel, and therefore, it cannot provide any evidence to directly support the theory of CRA stenosis resulting from mechanical compression by ODD. We could only speculate about this mechanism based on a character of blood flow changes in the CRA. Similarly, we were unable to distinguish whether the flow disturbances in the CRA had a primary character or occurred secondarily to the injury of the optic nerve fibers. Another potential limitation might be a selection bias, as our study included solely the patients with bilateral drusen. Therefore, we could not analyze the whole spectrum of hemodynamic changes in retrobulbar circulation associated with the presence of the drusen. Nevertheless, we used this criterion to obtain a large, homogenous patient population. Furthermore, we kept in mind that drusen occur bilaterally in most patients.

To summarize, this study provided a novel insight into the optic nerve hemodynamics in ODD. The study demonstrated that patients with bilateral ODD showed considerable disturbances of the CRA blood flow parameters. These disturbances might result from mechanical stenosis of the vessel caused by the drusen or might occur secondarily to the optic nerve atrophy. Further research is needed to verify whether the CRA flow disturbances progress with time and if patients with unilateral and bilateral drusen differ in terms of the retrobulbar hemodynamics.

## Data availability

The first author—Iwona Obuchowska has full access to all data in the study and she takes responsibility for the integrity of the data and accuracy of the data analysis.

## References

[1] Auw-Haedrich C, Staubach F, Witschel H (2002). Optic disc drusen. Surv. Ophthalmol..

[2] Schargus M, Gramer E (2008). Drusenpapille. Ophthalmologe.

[3] Spencer WH (1978). Drusen of the optic disc and aberrant axoplasmatic transport. The XXXIV Edward Jackson Memorial Lecture. Am. J. Ophthalmol..

[4] Tso MO (1981). Pathology and pathogenesis of drusen of the optic nerve head. Ophthalmology.

[5] Hamann S, Malmqvist L, Costello F (2018). Optic disc drusen: understanding an old problem from a new perspective. Acta. Opthalmol..

[6] Borruat FX, Sanders MD (1996). Vascular anomalies and complications of optic nerve drusen. Klin. Monbl. Augenheilkd..

[7] Farah SG, Mansour AM (1998). Central retinal artery occlusion and optic disc drusen. Eye.

[8] Kamath GG, Prasad S, Phillips RP (2000). Bilateral anterior optic neuropathy due to optic disc drusen. Eur. J. Ophthalmol..

[9] Purvin V, King R, Kawasaki A, Yee R (2004). Anterior ischaemic optic neuropathy in eyes with optic disc drusen. Arch. Ophthalmol..

[10] Munteanu M (2007). Hemorrhagic complications of drusen of the optic disk. J. Fr. Ophthalmol..

[11] Wilkins JM, Pomeranz HD (2004). Visual manifestations of visible and buried optic disc drusen. J. Neuroophthalmol..

[12] Lee AG, Zimmerman MB (2005). The rate of visual field loss in optic nerve head drusen. Am. J. Ophthalmol..

[13] Katz BJ, Pomeranz HD (2006). Visual field defects and retinal nerve fiber layer defects in eyes with buried optic nerve drusen. Am. J. Ophthalmol..

[14] Palmer E, Gale J, Crowston JG, Wells AP (2018). Optic nerve head drusen: an update. Neuro-Ophthalmol..

[15] Roh S (1998). Effect of optic nerve head drusen on nerve fiber layer thickness. Ophthalmology.

[16] Bernardczyk-Meller J, Wasilewicz R, Pecold-Stepniewska H, Wasiewicz-Rager J (2006). OCT and PVEP examination in eyes with visible optic disc drusen. Klin. Monbl. Augenheilkd..

[17] Malmqvist L, Wegener M, Sander BA, Hamann S (2016). Peripapillary retinal nerve fiber layer thickness corresponds to drusen location and extent of visual field defects in superficial and buried optic disc drusen. J Neuroophthalmol..

[18] Mullie MA, Sanders MD (1985). Scleral canal size and optic nerve head drusen. Am. J. Ophthalmol..

[19] Aumiller MS (2007). Optic disc drusen: complications and management. Optometry.

[20] Pinto LA, Vandewalle E, Marques-Neves C, Stalmans I (2014). Visual field loss in optic disc drusen patients correlates with central retinal artery blood velocity patterns. Acta Ophthalmol..

[21] Gosling R (1971). The quantitative analysis of occlusive peripheral arterial disease by a non-intrusive technique. Angiology.

[22] Bicer O, Atilla H (2019). Microvascular changes associated with optic disc drusen: case report. Turk. J. Ophthalmol..

[23] Flores-Reyes E, Hoskens K, Mansouri K (2017). Optic nerve head drusen: imaging using optical coherence tomography angiography. J. Glaucoma.

[24] Gaier ED, Rizzo JF, Miller JB, Cestari DM (2017). Focal capillary dropout associated with optic disc drusen using optical coherence tomographic angiography. J. Neuroophthalmol..

[25] Engelke H, Shajari M, Riedel J, Mohr N, Priglinger SG, Mackert MJ (2019). OCT angiography in optic disc drusen comparison with structural and functional parameters. Br. J. Ophthalmol..

[26] Cennamo G (2018). Optical coherence tomography angiography in optic nerve drusen. Ophthalmic Res..

[27] Quigley HA, Hofman RM, Addicks EM (1982). Quantitative study of optic nerve head capillaries in experimental optic disk pallor. Am. J. Ophthalmol..

[28] Rossitti S, Frisen L (1994). Remodeling of the retinal arterioles in descending optic atrophy following the principle of minimum work. Acta. Physiol. Scand..

[29] Frisen L, Claesson M (1984). Narrowing of retinal arterioles in descending optic atrophy: a quantitative clinical study. Ophthalmology.

[30] Sebag J, Feke GT, Delori FC (1985). Anterior optic nerve blood flow in experimental optic atrophy. Invest. Ophthalmol. Vis. Sci..

[31] Frisen L (2008). Evolution of drusen of the optic nerve head over 23 years. Acta. Ophthalmol..

